# Bisphenol A promotes cholesterol absorption in Caco-2 cells by up-regulation of NPC1L1 expression

**DOI:** 10.1186/s12944-016-0395-0

**Published:** 2017-01-06

**Authors:** Dan Feng, Jun Zou, Shanshan Zhang, Xuechun Li, Peiyang Li, Minqi Lu

**Affiliations:** 1 Guangdong Provincial Key Laboratory of Food, Nutrition and Health, Department of Preventive Medicine, School of Public Health, Sun Yat-sen University (Northern Campus), 74 Zhongshan Road 2, Guangzhou, Guangdong Province 510080 China; 2 Department of Cardiology, Affiliated NanHai Hospital of Southern Medical University, Foshan, 528200 China

**Keywords:** Bisphenol A, Cholesterol absorption, Caco-2 cells, Niemann-Pick C1-like 1, Sterol regulatory element binding protein-2, Hypercholesterolemia

## Abstract

**Background:**

Bisphenol A (BPA), an commonly exposed environmental chemicals in humans, has been shown to have a hypercholesterolemic effect with molecular mechanism not clear. Since intestinal cholesterol absorption plays a major role in maintaining total body cholesterol homeostasis, the present study is to investigate whether BPA affects cholesterol absorption in the intestinal Caco-2 cells. Methods: The Caco-2 cells were pretreated with BPA at different concentrations for 24 h and then incubated with radioactive micellar cholesterol for 2 h. The absorption of radioactive cholesterol was quantified by liquid scintillation. The expression of Niemann-Pick C1-like 1 (NPC1L1) and sterol regulatory element binding protein-2 (SREBP-2) was analyzed by Western blot and qPCR.

**Results:**

We found that confluent Caco-2 cells expressed NPC1L1, and the absorption of cholesterol in the cells was inhibited by ezetimibe, a specific inhibitor of NPC1L1. We then pretreated the cells with 0.1–10 nM BPA for 24 h and found that BPA at 1 and 10 nM doses promoted cholesterol absorption. In addition, we found that the BPA-induced promotion of cholesterol absorption was associated with significant increase in the levels of NPC1L1 protein and NPC1L1 mRNA. Moreover, the stimulatory effects of BPA on cholesterol absorption and NPC1L1 expression could be prevented by blockade of the SREBP-2 pathway.

**Conclusions:**

This study provides the first evidence that BPA promotes cholesterol absorption in the intestinal cells and the stimulatory effect of BPA is mediated, at least in part, by SREBP-2-NPC1L1 signaling pathway.

## Background

Hypercholesterolemia is a significant risk factor for cardiovascular disease [1]. In addition to denovo cholesterol synthesis, cholesterol derived from the dietary and biliary cholesterol absorption also contributes to the amount of cholesterol circulating in plasma [2]. In fact, the absorption of cholesterol from the intestine is an important determinant of plasma cholesterol levels [3], those individuals with high absorption rates are hypercholesterolemic and generally have an increased number of cardiovascular events [4]. Moreover, inhibition of cholesterol intestinal absorption leads to lower cholesterol levels [5].

Cholesterol absorption in intestine is a multi-step process and this process is mediated by a specific membrane protein named Niemann-Pick C1-like 1 (NPC1L1) protein [6], which can be specifically inhibited by ezetimibe [7]. NPC1L1 is highly expressed in the small intestine and it is required for intestinal cholesterol absorption. NPC1L1 knockout mice exhibited an 70% reduction in cholesterol absorption and were resistant to diet-induced hypercholesterolemia [6, 8]. Furthermore, NPC1L1 has been recently identified as a novel target gene of sterol regulatory element binding protein-2 (SREBP-2) [9]. There are two putative sterol regulatory elements in the human NPC1L1 promoter and they are essential for mediating the effects of cholesterol on promoter activity [9].

Bisphenol A (BPA) is a man-made compound highly prevalent in our environment and suspected to act as an endocrine disruptor [10]. It is intensively produced and used as a monomer of polycarbonate plastics and epoxy resins [11]. The human population is widely exposed to low levels of BPA, primarily by way of the diet by migration from food and beverage containers [11]. Biomonitoring surveys have demonstrated that detectable levels of BPA are present in the urine of nearly all sampled adults in U.S. population [12]. The ability of BPA to cause adverse human health effects is highly documented [13–15]. Many experimental and epidemiological studies highlighted potential links between BPA exposure and the development of cancer and disorders of reproductive, neuroendocrine, and immune systems [14, 16]. Recent epidemiological and animal studies have suggested that circulating BPA levels are associated with prevalence of coronary heart diseases and a number of cardiovascular risk factors such as hypercholesterolemia [17–19]. Several in vivo studies have shown that BPA exposure increased plasma total cholesterol and low density lipoprotein cholesterol levels [20, 21]. However, the molecular mechanism underlying the hypercholesterolemic effects are still unknown. Since the intestine plays a major role in maintaining total body cholesterol homeostasis, we, in the present study, addressed a question whether BPA affects the cholesterol absorption in the enterocytes.

## Methods

### Reagents

BPA and dimethyl sulfoxide (DMSO) were purchased from Sigma-Aldrich (St. Louis, MO, USA). The Dulbecco’s modified Eagle’s medium (DMEM), M199 medium, heat-inactivated fetal bovine serum (FBS),1% non-essential amino acids, and other materials were purchased from either Invitrogen (Carlsbad, CA, USA) or Sigma-Aldrich (St. Louis, MO, USA). [^14^C]cholesterol (50 mCi/mmol) was purchased from American Radiolabeled Chemicals Inc (St. Louis, MO, USA) and NPC1L1 antibody from Santa Cruz (Santa Cruz, CA, USA). cDNA synthesis kit was purchased from Invitrogen Life Technology (Carlsbad, CA, USA) and SYBR Green-based real-time PCR kit was obtained from Applied Biosystems (Foster City, CA,USA). All other chemicals, unless otherwise indicated, were purchased from Sigma-Aldrich (St. Louis, MO, USA).

### Cell culture and cholesterol absorption assay

Caco-2 cells were obtained from American Tissue Culture Collection (Virginia, USA) and were cultured in DMEM, containing 10% FBS, 1% penicillin-streptomycin, 2 mM L-glutamate, and 1% non-essential-amino acids to 100% confluence. Before the experiment of cholesterol absorption, the cells were firstly cultured in medium containing the delipidized FBS for 24 h as described [22]. Then the cells were washed three times with M199 buffer and incubated with fresh medium containing the cholesterol micelles for 2 h. The micellar cholesterol solutions were prepared as in our previous publication [22]. After incubation, the medium was removed, and the cells were washed three times with ice-cold PBS. The cell pellets were dissolved in 0.1 M NaOH, and an aliquot of 0.1 ml of the lysate was taken for liquid scintillation counting.

### Effects of BPA on cholesterol absorption

To investigate the effects of BPA on cholesterol absorption, the cells were pretreated with 0.1–10 nM BPA for 24 h [23]. After the pretreatment, the medium was removed and the cells were washed three times with ice-cold PBS, followed by incubation with the cholesterol micelles as described above. BPA was delivered to the cells using DMSO solvent, the amount of DMSO added to the cells was not greater than 0.1% (v/v). The cells treated with the equal amount of DMSO alone were taken as controls.

### Real-time quantitative PCR

Methods for RNA extraction and real-time quantitative PCR (qPCR) have been previously described in our previous publications [22]. The primers used in qPCR to quantify the mRNA of NPC1L1 and SREBP-2 were shown in Table 1. GAPDH was amplified as an internal control.

**Table 1:** Primers used in this study

Primer Name	Sequence
NPC1L1-F	5’-TATG GTCGCCCGAAGCA-3
NPC1L1-R	5’-TGCGGTTGTTCTGGAA ATACTG-3’
SREBP-2-F	5’-CAGCAGCCTTTGATATACCAGAATG -3’
SREBP-2-R	5’- AGGATGTCACCAGGCTTTGGAC -3’
GAPDH-F	5’-CATGAGAAGTATGACAACAGCCT-3’
GAPDH-R	5’-AGTCCTTCCACGATACCAAAGT-3’

### Western blot analysis

Methods for cell free extract and Western blotting has been previously described [22, 24]. In brief, 40 μg of proteins in cell lysate was subjected to 7.5% SDS-PAGE and transferred to a nitrocellulose membrane electrophoretically overnight. The membranes were incubated with anti-NPC1L1 antibody (1:5000) and then with second antibody (1:50000) conjugated with horseradish peroxidase. The specific NPC1L1 bands (145 kD) were identified by enhanced chemiluminescence advance reagent. The membranes were then stripped and re-probed with anti-actin antibody.

### RNA interference

SREBP-2 expression was knocked down by transfection of human SREBP-2 small interfering RNA (siRNA) with Caco-2 cells. A nonrelated, scrambled siRNA was used as a control. Transfection reagent and all siRNA oligos were designed and synthesized by Dharmacon. Transfections were carried out according to the manufacturer’s protocol using the DharmaFECT Reagent 4.

### Statistical analysis

The results are presented as mean ± S.E.M. Statistical analyses were performed using one-way analysis of variance (ANOVA) followed by the Bonferroni posttest for multiple comparisons. Differences were considered significant at *P* < 0.05.

## Results

### Absorption of cholesterol in Caco-2 cells was mediated by NPC1L1

**Fig. 1:**
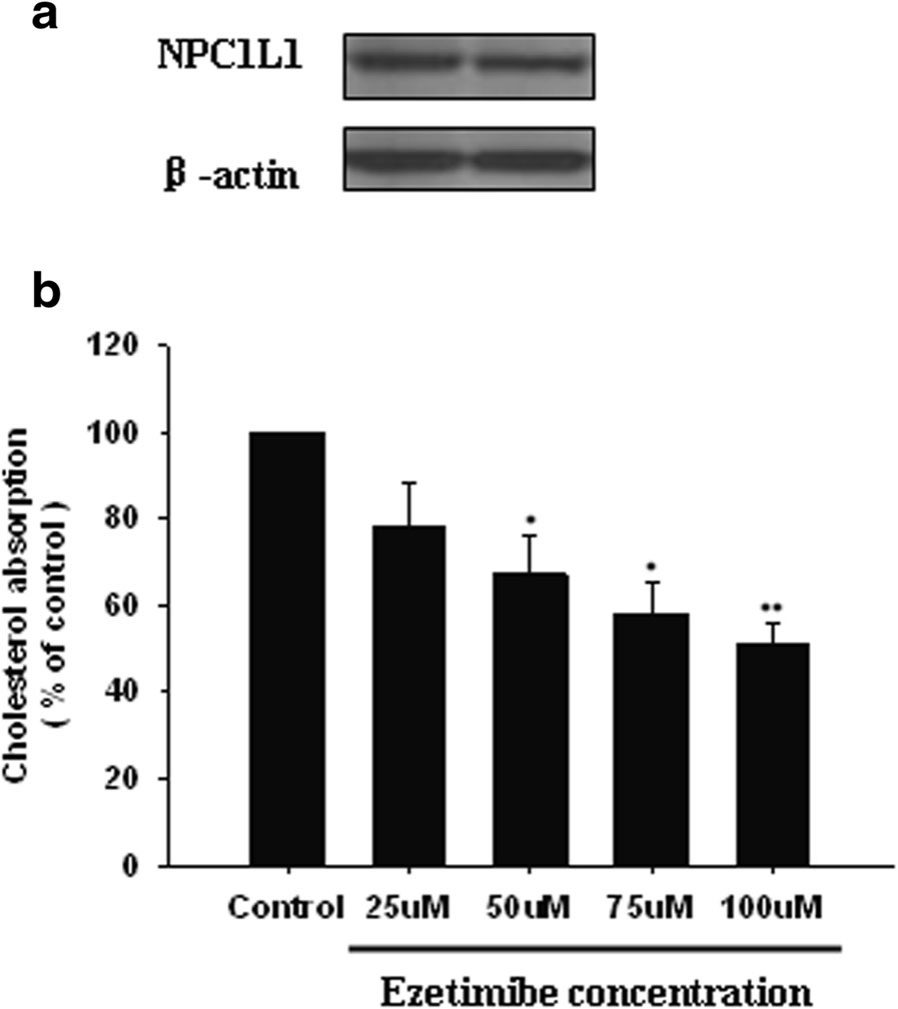
Cholesterol absorption by Caco-2 cells is mediated by NPC1L1. **a** The expression of NPC1L1 in Caco-2 cells. The cells were cultured to 100% confluence. The cellular protein was extracted and subjected to Western blot analysis for the expression of NPC1L1 and β-actin. **b** The cells were pretreated with ezetimibe at different concentrations for 2 h and then incubated with radioactive micellar cholesterol for 2 h. The absorption of cholesterol in the absence of ezetimibe was normalized to 100%. Results are mean ± SEM of three separate determinations. *P < 0.05, **P < 0.01

Because the cholesterol absorption in intestine was mediated by NPC1L1 and ezetimibe is a specific inhibitor for NPC1L1, we first examined whether the Caco-2 cells express NPC1L1 and whether cholesterol absorption in Caco-2 cells under the experimental conditions was related to the functions of NPC1L1. As shown in Fig. 1a, Western blot analysis clearly demonstrated the expression of NPC1L1 protein in these cells. In addition, the cholesterol absorption was dose-dependently inhibited by ezetimibe (Fig. 1b).

### BPA promoted cholesterol absorption in Caco-2 cells

**Fig. 2:**
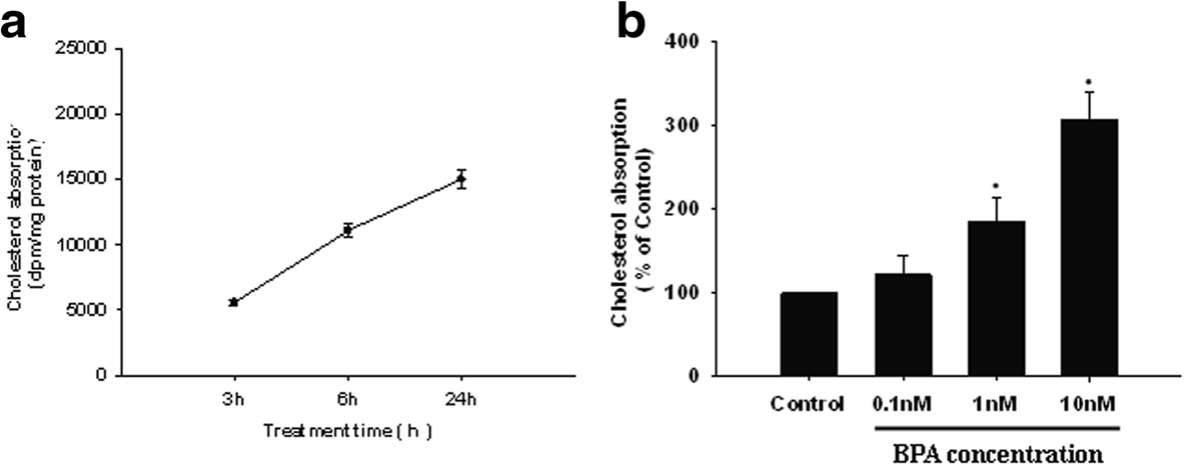
The effect of BPA on micellar cholesterol absorption in Caco-2 cells. **a** Time-dependent effect of BPA on cholesterol absorption in Caco-2 cells. The cells were pretreated with 10 nM BPA for different time, and then incubated with radioactive micellar cholesterol for 2 h. **b** Dose-dependent effect of BPA on cholesterol absorption in Caco-2 cells. The cells were pretreated with BPA at different concentrations for 24 h, and then incubated with radioactive micellar cholesterol for 2 h. The absorption of cholesterol was determined and that in the absence of BPA was taken as 100%. Results are mean ± SEM from triplicate determinations in three separate experiments. *P < 0.05 compared to untreated cells (Control)

We then addressed a question whether BPA can promote cholesterol absorption. Caco-2 cells were pretreated with 10 nM BPA for 3 h, 6 h and 24 h or pretreated with different concentrations of BPA for 24 h, then the amount of cholesterol absorption by the cells was then measured. The time-dependent effect of BPA on cholesterol absorption is presented in Fig. 2a, the results showed that the absorption of [^14^C] cholesterol was increased with the treantment time linearly and up to 24 h. Results from concentration dependent stimulation studies with BPA on the cellular absorption of [^14^C] cholesterol following 24 h of treatment are presented in Fig. 2b. As illustrated in this figure, there was no significant change in the cellular absorption of [^14^C] cholesterol in the presence of 0.1 nM of BPA (*P* > 0.05). The stimulatory effect of BPA started at concentrations ≥ 1 nM in a concentration dependent manner. The cellular absorption of cholesterol was significantly higher in the presence of 1 and 10 nM of BPA (*P* < 0.05) compared to control cells in the absence of BPA.

### BPA promoted NPC1L1 protein and mRNA expression

**Fig. 3:**
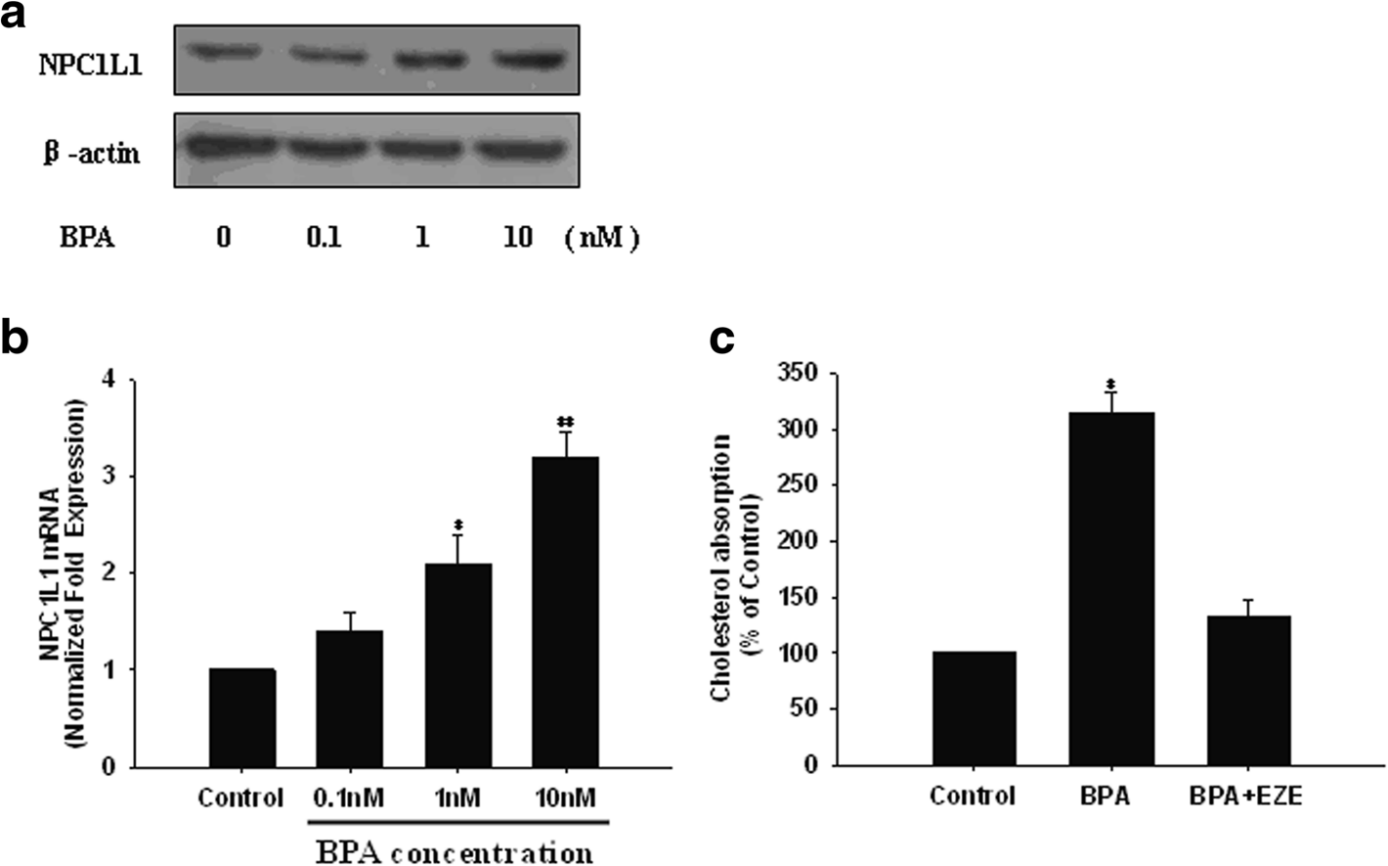
The effect of BPA on NPC1L1 expression in Caco-2 cells. **a** The cells were treated with BPA at different concentrations for 24 h, and the whole-cell lysates were analyzed by Western blot. The results are representative of three independent experiments. **b** NPC1L1 mRNA abundance was determined by real-time RT-PCR as described in Methods. Expression values were normalized to housekeeping genes, and expression in untreated cells was set to 1. Values shown represent means ± SEM of three independent experiments, *P < 0.05, **P < 0.01, compared to untreated cells. **c** After the blockade of NPC1L1 expression by ezetimibe(EZE), Caco-2 cells were incubated with BPA for 24 h, then incubated with radioactive micellar cholesterol for additional 2 h. The absorption of cholesterol in the absence of BPA was normalized to 100%. Results are mean ± SEM from triplicate determinations in three separate experiments. *P < 0.05 compared to untreated cell

To elucidate the molecular mechanism by which BPA promoted cholesterol absorption in Caco-2 cells, we then further analyzed the impact of BPA on NPC1L1 expression in the intestinal cells. After pretreating the cells with different concentrations of BPA for 24 h, the protein and mRNA expression of NPC1L1 were evaluated by Western blot and real-time PCR, respectively. As shown in Fig. 3a, pretreatment of the cells with BPA significantly increased NPC1L1 protein expression. No similar changes could be identified for the levels of β-actin. Simultaneously, the mRNA expression of NPC1L1 was dose-dependently increased by BPA, as normalized with that of control gene GAPDH (Fig. 3b). Furthermore, to address that the stimulatory effect of bisphenol A on cholesterol absorption is solely mediated by NPC1L1, we used NPCIL1 inhibitor ezetimibe to block NPC1L1 and found that the enhanced cholesterol absorption induced by bisphenol A was markedly attenuated (Fig. 3c).

### The SREBP-2 may be involved in the BPA-induced promotion of NPC1L1 expression and cholesterol absorption

**Fig. 4:**
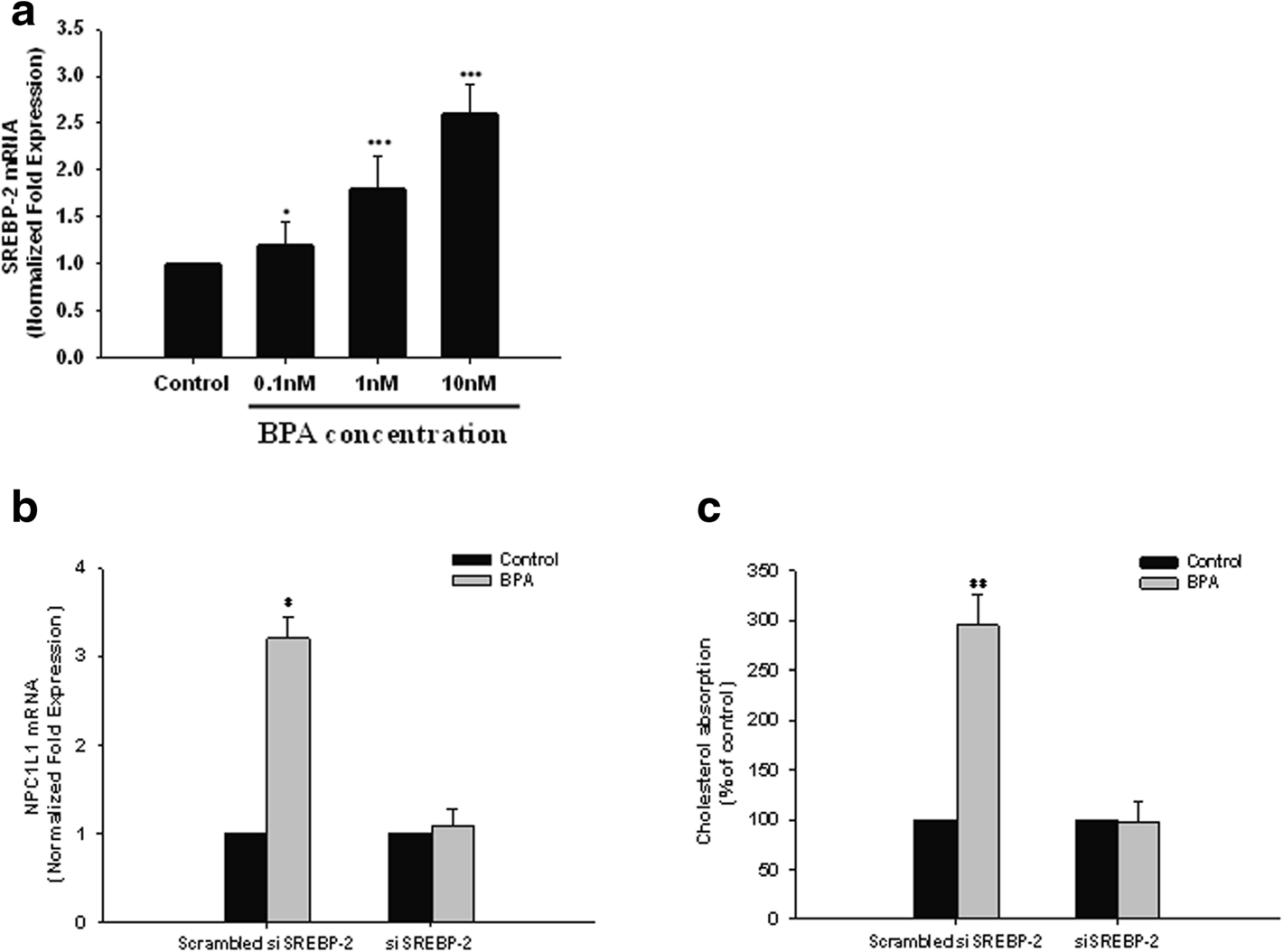
The promotion of BPA on NPC1L1 expression and cholesterol absorption may be mediated through SREBP-2. **a** Expression analysis of SREBP-2 in Caco-2 cells after 24 h of stimulation with increasing concentrations of BPA. Data are presented as means ± SEM of three independent experiments. *P < 0.05, ***P < 0.001 compared to untreated cells. **b** Up-regulation of NPC1L1 expression in Caco-2 cells after 24 h incubation with BPA, by transfecting short interfering RNA (siRNA) for SREBP-2 compared with scramble control transfections. Data are presented as means ± SEM of three independent experiments. *P < 0.05 compared to untreated cells. **c** After the inhibition of SREBP-2 expression using siRNA, Caco-2 cells were incubated with BPA for 24 h, then incubated with radioactive micellar cholesterol for additional 2 h. The absorption of cholesterol in the absence of BPA was normalized to 100%. Results are mean ± SEM from triplicate determinations in three separate experiments. **P < 0.01 compared to untreated cell

Since the expression of NPC1L1 is regulated by SREBP-2, we then wanted to know whether SREBP-2 could be involved in our observed NPC1L1 up-regulation. After pretreating the cells with BPA for 24 h, we found that the expression of SREBP-2 was dose-dependently up-regulated (Fig. 4a). We next examined the effects of the knockdown of SREBP-2 by small interfering RNA transfection on BPA-mediated NPC1L1 expression and cholesterol absorption. When SREBP-2 was suppressed by the specific RNA interference, no effect of BPA on NPC1L1 expression could be detected (Fig. 4b), and no significant increase of micellar cholesterol absorption by BPA in Caco-2 cells could be identified (Fig. 4c).

## Discussion

The findings of our present study demonstrate for the first time that BPA at environmentally relevant doses promotes cholesterol absorption in the intestinal cells and the stimulatory effect of BPA is mediated, at least in part, by SREBP-2-NPC1L1 signaling pathway.

BPA is an environmental chemical used as a constituent monomer in polycarbonate plastics, which are used extensively in drinks containers and food packaging, and in the production of oxidants used in the lining of canned goods [11]. Exposure to BPA is believed to be mainly through dietary intake with additional exposure through water, dental sealants, inhalation of household dusts, and exposure through skin [11]. A lot of studies have documented widespread human exposure to BPA [12]. Levels of BPA ranging from 0.3 to 5 ng/mL (~1–20 nM) are present in adult and fetal human plasma, urine, and breast milk [25]. BPA, a lipophilic compound, can accumulate in fat, with detectable levels found in 50% of breast adipose tissue samples from women [26]. BPA expoure has been reported to cause adverse human health effects such as cancer, reproductive, neuroendocrine, and immune systems disorders [13, 14, 16]. Additionally, several lines of recent evidence suggest that an association between circulating BPA levels and hypercholesterolemia, studies in animals and humans have also shown that BPA exposure increased cholesterol levels in blood [19–21], but the molecular mechanisms underlying the hypercholesterolemic effects of BPA are still unkown. Since the intestine plays a major role in maintaining total body cholesterol homeostasis, and human exposure to BPA is primarily through dietary intake, whether absorption of cholesterol in the enterocytes can be affected by BPA is therefore an important question to be studied. Our results for the first time show that BPA at environmentally relevant doses has stimulatory effects on cholesterol absorption in the intestinal cells. The conclusion is supported by the following evidence. First, the Caco-2 cells used in our experiments expressed NPC1L1 and the absorption of cholesterol in our system was mainly mediated by NPC1L1. Second, pretreatment of the cells with BPA significantly promoted cholesterol absorption, which was accompanied by an increase of NPC1L1 protein and mRNA expression.

Exposure of humans to BPA is ubiquitous at low levels(~1–20 nM), the dose used in the present study is environmentally relevant and our research may have clinical implication. Several studies have revealed that exposure to environmentally relevant BPA doses could alter various biological functions, including reproductive, behavioral, metabolic, and immune systems [16]. Hugo et al. has shown that BPA at environmentally relevant doses inhibits the release of a key adipokine that protects humans from metabolic syndrome [23], which may be one mechanism that is responsible for the metabolic disorders and obesity caused by BPA. Furthermore, our results also potentially suggest a new mechanism that is responsible for the hypercholesterolemic effects of BPA. It is noted that the liver is also important for systemic lipid metabolism and is the primary target organ of BPA. A recent study in mice has shown that BPA exposure significantly increased the expression levels of genes related to lipid synthesis such as HMG-CoA reducase, the key enzyme responsible for cholesterol synthesis in liver [27]. Take together, there results indicate that BPA can increase the cholesterol levels by two mechanisms, one in the intestine to enhance the absorption via NPC1L1 transporter, and the other in the liver to increase the synthesis by HMG-CoA reducase.

NPC1L1 is a crucial transporter for cholesterol absorption [6]. Understanding the regulation of its expression is of importance in human health and disease. Recent studies have shown the increase in NPC1L1 expression in hypercholesterolemia associated with diseases such as diabetes mellitus [28, 29]. Moreover, NPC1L1 inhibition or deficiency was shown to protect against diet-induced hypercholesterolemia and hepatic steatosis [30, 31]. Since NPC1L1 plays a pivotal role in mediating intestinal cholesterol absorption and the processes of cholesterol homeostasis, we argued that BPA may directly influence its function. In our studies, we found that pretreatment of the cells with BPA significantly increased NPC1L1 mRNA and protein levels, and the changes in cholesterol absorption consistently corresponded to the changes in NPC1L1 mRNA and protein expression. Moreover, to address that the stimulatory effect of bisphenol A on cholesterol absorption is solely mediated by NPC1L1, we further used NPCIL1 inhibitor ezetimibe to block NPC1L1 and found that the enhanced cholesterol absorption induced by bisphenol A was markedly attenuated. These results suggest the mechanism may involve an increase in cholesterol absorption due to a up-regulation of NPC1L1, thus contributing to the hypercholesterolemic effect of BPA.

Regarding the molecular signals regulating the intestinal expression of NPC1L1, we found that expression of the transcription factor SREBP-2 is involved in BPA-induced up-regulation of NPC1L1. It is known that the expression of NPC1L1 is mainly regulated by transcription factor SREBP-2. There are two putative sterol regulatory elements in the human NPC1L1 promoter [9], and the expression of NPC1L1 gene can be up-regulated by SREBP-2 activation in the intestine [9]. Moreover, BPA has been found to induce liver SREBP-2 expression in mice [20, 32]. Thus, we hypothesized that BPA influenced NPC1L1 expression presumably through the SREBP-2-mediated signal transduction pathway. In supporting this hypothesis, when Caco-2 cells were stimulated with BPA, we did find significantly increased expression of the SREBP-2 in the cells, and when SREBP-2 was depleted by siRNA, NPC1L1 expression was no longer increased by BPA, and no increase in micellar cholesterol absorption was observed. Our studies demonstrate that BPA modulates intestinal NPC1L1 expression at both transcriptional and translational regulations and the SREBP-2 transcription factor may be critically involved in such regulations.

Furthermore, with regard to the mechanism by which BPA up-regulated the expression of SREBP-2, the epigenetic regulation may be involved. It is known that promoter methylation levels tend to relate negatively to gene expression. Ke et al. has recently shown that the promoters of SREBP-2 and HMG-CoA reducase were hypomethylated in the BPA-exposed mice, which is very likely to contribute to the promoted transcription of SREBP-2 and its targets. Because DNA methyltransferase knockdown led to the promoter hypomethylation and increased mRNA expression of SREBP-2 and HMG-CoA reducase [27]. These results indicated that SREBP-2 was up-regulated by BPA via reprogramming the DNA methylation patterns.

## Conclusions

In conclusion, our current results imply a potential role of BPA, at environmentally relevant concentrations that can be detected in vivo, in stimulating intestinal cholesterol absorption and in promoting hypercholesterolaemia. The mechanisms may be involved in SREBP-2 activation and subsequent up-regulation of NPC1L1 expression.

## Abbreviations

BPA: Bisphenol A
DMEM: Dulbecco’s modified Eagle’s medium
DMSO: Dimethyl sulfoxide
FBS: Fetal bovine serum
NPC1L1: Niemann-Pick C1-like 1
qPCR: quantitative PCR
siRNA: short interfering RNA
SREBP-2: Sterol regulatory element binding protein-2

## References

[1] Weingartner O, Lutjohann D, Bohm M, Laufs U (2011). Cholesterol homeostasis and cardiovascular risk. Dtsch Med Wochenschr.

[2] Howles PN (2016). Cholesterol absorption and metabolism. Methods Mol Biol.

[3] Kruit JK, Groen AK, van Berkel TJ, Kuipers F (2006). Emerging roles of the intestine in control of cholesterol metabolism. World J Gastroenterol.

[4] Stitziel NO, Won HH, Morrison AC, Peloso GM, Do R (2014). Inactivating mutations in NPC1L1 and protection from coronary heart disease. N Engl J Med.

[5] Smith BA, Wright C, Davidson M (2015). Role of Ezetimibe in lipid-lowering and cardiovascular disease prevention. Curr Atheroscler Rep.

[6] Altmann SW, Davis HR, Zhu LJ, Yao X, Hoos LM (2004). Niemann-Pick C1 Like 1 protein is critical for intestinal cholesterol absorption. Science.

[7] Garcia-Calvo M, Lisnock J, Bull HG, Hawes BE, Burnett DA (2005). The target of ezetimibe is Niemann-Pick C1-Like 1 (NPC1L1). Proc Natl Acad Sci US A..

[8] Davis HR, Hoos LM, Tetzloff G, Maguire M, Zhu LJ (2007). Deficiency of Niemann-Pick C1 Like 1 prevents atherosclerosis in ApoE-/- mice. Arterioscler Thromb Vasc Biol.

[9] Alrefai WA, Annaba F, Sarwar Z, Dwivedi A, Saksena S (2007). Modulation of human Niemann-Pick C1-like 1 gene expression by sterol: Role of sterol regulatory element binding protein 2. Am J Physiol Gastrointest Liver Physiol.

[10] Corrales J, Kristofco LA, Steele WB, Yates BS, Breed CS (2015). Global assessment of bisphenol A in the environment: review and analysis of its occurrence and bioaccumulation. Dose Response.

[11] Vandenberg LN, Hauser R, Marcus M, Olea N, Welshons WV (2007). Human exposure to bisphenol A (BPA). Reprod Toxicol.

[12] Calafat AM, Ye X, Wong LY, Reidy JA, Needham LL (2008). Exposure of the U.S. population to bisphenol A and 4-tertiary-octylphenol: 2003-2004. Environ Health Perspect.

[13] Rezg R, El-Fazaa S, Gharbi N, Mornagui B (2014). Bisphenol A and human chronic diseases: current evidences, possible mechanisms, and future perspectives. Environ Int.

[14] Rochester JR (2013). Bisphenol A, and human health: a review of the literature. Reprod Toxicol.

[15] Srivastava S, Gupta P, Chandolia A, Alam I (2015). Bisphenol A: a threat to human health?. J Environ Health.

[16] Richter CA, Birnbaum LS, Farabollini F, Newbold RR, Rubin BS, Talsness CE (2007). In vivo effects of bisphenol A in laboratory rodent studies. Reprod Toxicol.

[17] Melzer D, Osborne NJ, Henley WE, Cipelli R, Young A (2012). Urinary bisphenol A concentration and risk of future coronary artery disease in apparently healthy men and women. Circulation.

[18] Kim MJ, Moon MK, Kang GH, Lee KJ, Choi SH (2014). Chronic exposure to bisphenol A can accelerate atherosclerosis in high-fat-fed apolipoprotein E knockout mice. Cardiovasc Toxicol.

[19] Olsen L, Lind L, Lind PM (2012). Associations between circulating levels of bisphenol A and phthalate metabolites and coronary risk in the elderly. Ecotoxicol Environ Saf.

[20] Marmugi A, Lasserre F, Beuzelin D, Ducheix S, Huc L (2014). Adverse effects of long-term exposure to bisphenol A during adulthood leading to hyperglycaemia and hypercholesterolemia in mice. Toxicology.

[21] Moghaddam HS, Samarghandian S, Farkhondeh T (2015). Effect of bisphenol A on blood glucose, lipid profile and oxidative stress indices in adult male mice. Toxicol Mech Methods.

[22] Zou J, Feng D (2015). Lycopene reduces cholesterol absorption through the downregulation of Niemann-Pick C1-like 1 in Caco-2 cells. Mol Nutr Food Res.

[23] Hugo ER, Brandebourg TD, Woo JG, Loftus J, Alexander JW (2008). Bisphenol A at environmentally relevant doses inhibits adiponectin release from human adipose tissue explants and adipocytes. Environ Health Perspect.

[24] Feng D, Ohlsson L, Duan RD (2010). Curcumin inhibits cholesterol uptake in Caco-2 cells by down-regulation of NPC1L1 expression. Lipids Health Dis.

[25] Welshons WV, Nagel SC, vom Saal FS (2006). Large effects from small exposures. III. Endocrine mechanisms mediating effects of bisphenol A at levels of human exposure. Endocrinology.

[26] Fernandez MF, Arrebola JP, Taoufiki J, Navalon A, Ballesteros O (2007). Bisphenol-A and chlorinated derivatives in adipose tissue of women. Reprod Toxicol.

[27] Ke ZH, Pan JX, Jin LY, Xu HY, Yu TT (2016). Bisphenol A exposure may induce hepatic lipid accumulation via reprogramming the DNA methylation patterns of genes involved in lipid metabolism. Sci Rep.

[28] Lally S, Owens D, Tomkin GH (2007). Genes that affect cholesterol synthesis, cholesterol absorption, and chylomicron assembly: the relationship between the liver and intestine in control and streptozotosin diabetic rats. Metabolism.

[29] Lally S, Tan CY, Owens D, Tomkin GH (2006). Messenger RNA levels of genes involved in dysregulation of postprandial lipoproteins in type 2 diabetes: the role of Niemann-Pick C1-like 1, ATP-binding cassette, transporters G5 and G8, and of microsomal triglyceride transfer protein. Diabetologia.

[30] Davies JP, Scott C, Oishi K, Liapis A, Ioannou YA (2005). Inactivation of NPC1L1 causes multiple lipid transport defects and protects against diet-induced hypercholesterolemia. J Biol Chem.

[31] Deushi M, Nomura M, Kawakami A, Haraguchi M, Ito M (2007). Ezetimibe improves liver steatosis and insulin resistance in obese rat model of metabolic syndrome. FEBS Lett.

[32] Marmugi A, Ducheix S, Lasserre F, Polizzi A, Paris A (2012). Low doses of bisphenol A induce gene expression related to lipid synthesis and trigger triglyceride accumulation in adult mouse liver. Hepatology.

